# Mechanisms of resistance to mitochondria-targeted therapy in pancreatic cancer

**DOI:** 10.18632/oncotarget.27976

**Published:** 2021-08-03

**Authors:** Stepana Boukalova, Zuzana Ezrova, Jiri Neuzil

**Keywords:** complex I inhibitors, transforming growth factor β, SMAD4, pancreatic cancer, drug resistance

Pancreatic cancer remains a major unsolved health problem as a result of limited success rate of chemo- or radiation therapy. New treatment strategies are urgently needed to improve the survival rate of patients with this type of malignancy. Mitochondrial energy metabolism is currently recognized as a promising treatment target in a wide spectrum of cancers. Recent studies demonstrated that mitochondrial oxidative phosphorylation (OXPHOS) is vital for tumor cells not solely as a source of ATP, but even more importantly, to supply building blocks for their anabolic needs [1, 2]. A number of drugs targeting respiratory chain complexes, particularly complex I (CI) inhibitors, are being evaluated in clinical trials for different types of neoplastic diseases, including pancreatic cancer [3, 4]. Among them, metformin, a biguanide derivative, has attracted considerable attention since it is already used in the clinic for treatment of type 2 diabetes. Furthermore, mitochondrially targeted metformin (MitoMet) was developed to augment the therapeutic activity of this mild CI inhibitor. This modification resulted in markedly increased efficacy to target mitochondrial CI and in elevated tumor-suppressing effects in mouse models of pancreatic cancer [5, 6]. However, due to considerable genetic as well as metabolic heterogeneity of pancreatic tumors, determining the factors governing responsiveness to complex I inhibitors is of great importance in order to select patients who will best benefit from this type of therapy.

Ezrova *et al*. found a positive correlation between susceptibility to CI inhibitors from the biguanide class and expression of a tumor suppressor gene, SMAD4, in pancreatic ductal adenocarcinoma (PDAC) cell lines [7]. SMAD4, a key mediator of the transforming growth factor β (TGFβ) pathway, is inactivated in more than 50% of advanced-stage PDAC cases, which has been linked to increased aggressiveness of the disease [8]. By investigating the mechanism of resistance to biguanides in SMAD4-negative PDAC cells, Ezrova *et al*. found that SMAD4 loss markedly influenced mitochondrial function and morphology. Deletion of SMAD4 resulted in mitochondrial fragmentation, altered cristae morphology, and decreased respiration in the cells. Interestingly, similar mitochondrial alterations were found in SMAD4-positive cells exposed to TGFβ, and this was linked to decreased responsiveness to biguanides, too [7]. This is an apparently contradictory result, in particular considering the fact that SMAD4 inactivation abrogates canonical TGFβ signaling. The mechanism by which SMAD4 loss mimics the TGFβ-activated state needs further investigation. It may be associated with the MAPK/ERK pathway, a downstream target of TGFβ signaling, which was found to be basally activated in SMAD4-deficient PDAC cells. Increased MAPK/ERK signaling in SMAD4-null cells could be mediated via upregulation of non-canonical SMAD4-independent TGFβ signaling, however, other cellular pathways may be involved as well.

Fragmentation of the mitochondrial network is associated with malignant transformation of cancer cells, and it was shown to be involved in metastatic dissemination [9]. Since both TGFβ and SMAD4 loss promote metastasis in PDAC [8], we can speculate that mitochondrial remodeling may be (at least partially) responsible for the malignant phenotype. The pro-metastatic activity of TGFβ is traditionally attributed to activation of epithelial-mesenchymal transition (EMT), a process that promotes cell migration [8]. TGFβ-induced EMT is largely dependent on functional SMAD4, however, SMAD4 suppression was found to be associated with the mesenchymal phenotype in PDAC as well [7, 8, 10]. This paradoxical role of SMAD4 deficiency could be explained by its effects on mitochondrial morphology and function, which may underlie the metastatic transformation of PDAC.

Although mitochondrial function is comparably altered by TGFβ signaling and SMAD4 loss, Ezrova *et al*. showed that resistance to biguanides is mediated via different mechanisms under these conditions [7]. In SMAD4-deficient cells, resistance to MitoMet is linked to elevated level of mitochondria-specific autophagy, *i.e*., mitophagy. The increased mitophagic flux in SMAD4-null cells is at least partially mediated via basally activated MAPK/ERK signaling, pointing to an important role of this pathway in resistance to CI inhibitors and possibly other effects promoted by SMAD4 loss [11]. On the other hand, activation of TGFβ signaling in SMAD4-proficient cells is not associated with elevated mitophagic flux, even though general autophagy is increased. Besides, inhibition of autophagy has no effect on sensitivity to MitoMet. Instead, TGFβ-induced resistance was found to be in control of activated EMT program. This is in line with the study of Zheng *et al*., who found that EMT signaling decreases vulnerability to anti-proliferative drugs in pancreatic cancer [12].

These findings suggest that PDAC patients could benefit from therapy combining a metformin derivative with a TGFβ signaling blocker in case of SMAD4-positive tumors, or inhibitor of autophagy in SMAD4-negative PDAC cases. Of note, a highly potent CI inhibitor – MitoTam (*i.e*., mitochondrially targeted tamoxifen), overcomes the resistance induced both by TGFβ signaling and SMAD4 loss, which makes it an intriguing candidate for PDAC therapy. The highly hydrophobic nature of MitoTam, which allows its rapid accumulation in mitochondria, was proposed to account for the different mode of action of this agent compared to biguanides. MitoTam was shown to promptly increase reactive oxygen species (ROS) production leading to eradication of cancer cells via apoptosis [13]. On the other hand, biguanides act more slowly, whereby their anti-tumor activity may be suppressed by cellular protective mechanisms, for instance by increased mitophagy. MitoTam is currently being evaluated in a clinical trial in patients with metastatic progressive solid tumors of different types with promising initial results. Its testing in PDAC patients may bring the desired progress in pancreatic cancer therapy.

Although targeting mitochondrial oxidative phosphorylation has brought encouraging results in pre-clinical cancer models, the clinical development of inhibitors of respiratory complexes is in its infancy. It is critically important to fine-tune the biochemical and pharmacological properties of these drugs to maximize the anti-tumor activity, while keeping the adverse effects to minimum. On top of that, we need to understand the mechanisms that may underlie the resistance to this type of therapy and determine candidates for combinatorial therapy that would not only be more potent, but also minimize the risk of resistance. In case of PDAC, targeting autophagy or TGFβ signaling might be advantageous when combined with biguanide therapy.
